# Editorial: Cerebral visual impairment, visual development, diagnosis, and rehabilitation

**DOI:** 10.3389/fnhum.2022.1057401

**Published:** 2022-11-15

**Authors:** Corinna M. Bauer, Arlette J. van Sorge, Richard Bowman, Frouke N. Boonstra

**Affiliations:** ^1^Lab of Neuroimaging and Vision Science, Gordon Center for Medical Imaging, Department of Radiology, Massachusetts General Hospital, Boston, MA, United States; ^2^Department of Ophthalmology, Massachusetts Eye and Ear, Harvard Medical School, Boston, MA, United States; ^3^Royal Dutch Visio, National Foundation for the Visually Impaired and Blind, Huizen, Netherlands; ^4^Ophthalmology Department, Great Ormond Street Hospital NHS Foundation Trust, London, United Kingdom; ^5^Developmental Neurosciences, UCL Great Ormond Street Institute of Child Health, London, United Kingdom; ^6^London School of Hygiene and Tropical Medicine, University of London, London, United Kingdom; ^7^Department of Cognitive Neuroscience, Donders Institute for Brain, Cognition and Behavior, Radboud University Medical Centre Nijmegen, Nijmegen, Netherlands; ^8^Behavioral Science Institute, Radboud University, Nijmegen, Netherlands

**Keywords:** cerebral visual impairment, visual development, diagnosis, neuroophthalmology, pediatric ophthalmology, neurodevelopmental disorder, visual perceptual disorder, cortical visual impairment

## Introduction

Cerebral (or cortical) visual impairment (CVI) is a verifiable visual dysfunction(s) that cannot be attributed to any potentially co-occurring ocular condition (Sakki et al., 2018). CVI can manifest in many ways including varying degrees of reduced visual function (i.e., acuity, fields, etc.), as well as visual perceptual functions depending on which part of the brain is impacted (Chokron et al.). Despite the potential long-term ramifications on a child's development and quality of life, there remains an urgent need for evidence-based assessments, diagnostic protocols, and interventions for children with CVI. This special issue includes 18 manuscripts from which three major themes emerged: 1. Current challenges in receiving an accurate and timely diagnosis of CVI, 2. Strides toward identifying risk factors, screening tools, and biomarkers for the multidisciplinary diagnosis of CVI across multiple age ranges, and 3. The potential impact of interventions on outcomes and quality of life in children affected by CVI.

## Theme 1: Current challenges in receiving an accurate and timely diagnosis of CVI

As highlighted by Chokron et al., the prevalence of CVI is increasing worldwide, including industrialized and developing nations. Despite the increasing recognition of CVI, distinguishing the consequences of visual dysfunctions from neurodevelopmental conditions, including autism spectrum disorder, attention deficit disorder, and dyslexia, remains challenging. Consequently, the aberrant behaviors may not be recognized as being due to a visual disorder and may therefore be misdiagnosed. The authors highlight the need to provide additional information and training to clinicians for differentiating CVI from other neurodevelopmental or specific learning disorders.

Chokron et al. also provide compelling evidence for the negative consequences that CVI can have on development, including motor function, social interaction, and learning. A comprehensive multidisciplinary assessment may assist to optimally plan intervention and habilitative strategies, which might afford additional opportunities for neuroplasticity. The deleterious effects of a delayed diagnosis are further presented in the study by Goodenough et al.. Through a series of semi-structured qualitative interviews the authors probe how CVI impacts all aspects of everyday life. These interviews emphasize how receiving a diagnosis of CVI can substantially transform the child's ability to access their environment. However, CVI may fail to be recognized, preventing the patient from accessing supports and services for the visually impaired. Recently published guidelines may be useful to gain access to supports (Boonstra et al.).

## Theme 2: Strides toward identifying risk factors, screening tools, and biomarkers for the multidisciplinary diagnosis of CVIs

Historically, CVI has been mainly a diagnosis of exclusion, with no standardized diagnostic procedures and guidelines. Subsequently, Boonstra et al. performed five literature searches to determine the level of evidence supporting the use of specific assessments for the diagnosis and referral in CVI. The outcome of searches on medical history and the use of questionnaires, as well as ophthalmological, neuropsychological, neurological, and genetic assessments are presented. These outcomes suggest that specific evaluations can be used to identify those with CVI or at risk for CVI, depending upon the individual patient's age and developmental level.

Parents of children with CVI frequently report a desire for early screening and diagnosis. Along these lines, Kooiker et al. investigated whether it was effective to screen 1 year old preterm children for visual processing dysfunctions (VPD) using neurological history and eye tracking. At 1 year of age, 38% of the children examined were at risk for a VPD, with an increase in abnormal visual orienting functions at the age of 2 years, suggesting that some individuals demonstrate a delayed onset of their visual dysfunctions. On the other hand, evidence from Galli et al., suggests that in children with cerebral palsy (CP), signs of CVI may be more common in younger children as compared to older children. However, this may be in part due to differences in the specific CVI assessments in each study, with the later focusing more on ophthalmological characteristics as opposed to visual perception in the study of Kooiker et al.. Additionally, Wilton et al. provide evidence for potential CVI in children with Down Syndrome, suggesting the need for screening in neurodevelopmental and genetic disorders. Together, these studies provide further evidence supporting the need for early and repeated screening of high risk groups for CVI (including preterm birth, CP, and neurodevelopmental and genetic disorders) before or until at least school age, so that no child is mis- or un-diagnosed and can get the support needed early in life.

Reduced visual acuity and abnormal looking patterns are often the first signs in children that warrant a visual examination by an eye care professional. In their retrospective chart review study, Raja et al. sought to determine whether the discrepancy in visual acuity as measured by preferential looking tests (PLT) and visual evoked potentials (VEP) could serve as a potential biomarker for CVI. The results suggest that VEP acuity exceeding PLT acuity by one or more octaves may be a biomarker for CVI, although this needs to be confirmed in a prospective study in a secondary sample.

It is increasingly recognized that children with normal or near normal visual acuity can have a diagnosis of CVI and present with higher visual dysfunctions as a consequence of brain injury, maldevelopment, or genetic disorders, as well as other causes (e.g., Chokron et al.; Chandna et al.). Unfortunately, CVI in children with good visual acuity often remains undiagnosed. Chandna et al. sought to determine the spectrum of higher order visual dysfunctions in children with CVI and good visual acuity using a 51-item inventory. They propose a subset of 11 questions that may be particularly discriminating for identifying CVI in patients with good visual acuity.

Individuals with CVI frequently demonstrate increased visual latency, requiring more time to perform visual tasks (Barsingerhorn et al., 2018, 2019). In this issue, Tanke et al. investigated the use of the developmental eye movement test (DEM) as a diagnostic aid for CVI. They suggested that, in combination with crowding assessment, the DEM may be a useful addition to the assessment battery.

One of the higher order visual dysfunctions commonly seen in CVI involves motion perception. New evidence from van der Zee et al., suggests that children with brain damage may be at an increased risk of isolated and combined motion perception problems, including global motion, speed of motion, or motion-defined form and this was independent of cognition.

In another article, van der Zee et al. also reported on the correspondence between dorsal and ventral stream dysfunctions, finding a higher proportion of dorsal stream dysfunctions (e.g., challenges with motion perception, visual attention, and visuomotor tasks) in those presenting with ventral stream dysfunctions (e.g., object recognition) as measured by the L94 (as compared to those without object recognition impairments). Of the dorsal stream dysfunctions evaluated, motion perception, and visual attention were more frequently impacted than visuomotor skills. Together, these studies suggest that evaluations should include at minimum an ophthalmological assessment, as well as evaluations of both dorsal and ventral stream visual functions.

The value of MRI in the diagnostic process remains contentious. Sakki et al. sought to determine the association between brain lesions visible on MRI and the level of visual dysfunction in two subgroups of patients with CVI. No anatomical correlates with specific visual dysfunctions were identified, but the authors concluded that neuroimaging may prove valuable for assisting in the diagnosis and identification of those at risk for CVI due to brain injury to the visual processing networks in the brain.

Additionally, ocular imaging tools may be useful for investigating CVI. Lennartsson et al. sought to determine the relationship between brain injury and retinal degeneration. They reported on differential patterns of visual field restriction and OCT (optical coherence tomography of optic nerve head) found across subgroups of CVI, corresponding to diffusion tractography measures.

Eye tracking is becoming increasingly implemented as a potential diagnostic aid for CVI, particularly as it can be implemented in patients with limitations in mobility or verbal communication. In addition to the article by Kooiker et al., eye tracking technology was also used by Mooney et al. in their study. Their “visual ladder” approach for detecting, quantifying, and comparing eye movements may enable a robust and rapid quantification of visual impairment in patients with CVI, including those who have limited verbal abilities. This is important because it is only through objective, quantifiable measurement that one can determine the impact of rehabilitation strategies.

Another feasibility study in this special issue by Almagati and Kran describes a method combining synchronous (remote) and asynchronous assessment and data collection in a pediatric low vision clinic setting. The asynchronous components included recruitment, pre-assessment information, the Flemish CVI questionnaire, Vineland-3 comprehensive parent questionnaire for assessment of age equivalent, and vision function tests, such as contrast sensitivity. The synchronous components were administered *via* Zoom telehealth and included assessment of visual acuity *via* FrACT electronic software and assessment of visual perceptual batteries *via* the CVIT 3-6. This hybrid approach may prove beneficial for both the clinic and research setting, particularly when evaluating individuals who are physically remote from the clinic/research site. They also demonstrate that this approach is indeed possible in the CVI population.

## Theme 3: The impact of interventions in CVI

Two articles in this issue focused on a longitudinal evaluation of CVI outcomes following intervention. The first, by Jimenez-Gomez et al., was a retrospective chart review. Their goal was to identify outcome predictors for CVI severity as well as factors associated with a change in their grading scale (based on visual function). The majority of patients in this study had limited functional use of vision (they demonstrated no blink to light or could not fixate and follow), limiting the generalizability of the results across the CVI spectrum.

The effect of intervention was also evaluated by Duke et al.. Their randomized clinical trial sought to determine whether individualized visual support strategies, derived from the insight question inventory, a structured history tool as also investigated by Chandna et al., could improve quality of life outcomes beyond that observed in standard therapy. The results suggest that, although there was no change in generic QoL scores, there was a small but significant improvement in speech and communication subscale following a 6-week intervention period. Further research in this area over a longer timeframe is required.

## Conclusion

This Research Topic provides new evidence supporting the diagnostics and rehabilitation of children with CVI. New techniques such as time-related tests with optotypes, eye tracking, OCT, and MRI have been useful to gather evidence in children with multiple disabilities. A more standardized multidisciplinary battery of assessments that may be used for screening, assessment, and diagnosis of CVI across various underlying conditions will result from further investigations. Applied research on visual acuity measurement appropriate to developmental age as well as other objective measures, such as visual fields, OCT, and MRI, will enable us to increase diagnostic possibilities such that no child with CVI is left undiagnosed and left without access to services in order that they may reach their full potential.

## Author contributions

All authors listed have made a substantial, direct, and intellectual contribution to the work and approved it for publication.

## Conflict of interest

The authors declare that the research was conducted in the absence of any commercial or financial relationships that could be construed as a potential conflict of interest.

## Publisher's note

All claims expressed in this article are solely those of the authors and do not necessarily represent those of their affiliated organizations, or those of the publisher, the editors and the reviewers. Any product that may be evaluated in this article, or claim that may be made by its manufacturer, is not guaranteed or endorsed by the publisher.
